# New approaches in childhood asthma treatment

**DOI:** 10.1097/ACI.0000000000000922

**Published:** 2023-06-23

**Authors:** Riccardo Castagnoli, Ilaria Brambilla, Mattia Giovannini, Gian Luigi Marseglia, Amelia Licari

**Affiliations:** a Pediatric Unit, Department of Clinical, Surgical, Diagnostic and Pediatric Sciences, University of Pavia; b Pediatric Clinic, Fondazione IRCCS Policlinico San Matteo, Pavia; c Allergy Unit, Meyer Children's Hospital IRCCS; d Department of Health Sciences, University of Florence, Florence, Italy

**Keywords:** asthma, biologics, children, therapy

## Abstract

This review aims to summarize the most recent advances in asthma management, focusing on novel approaches to pediatric asthma.

**Recent findings:**

In recent years, the therapeutic tools for pediatric asthma have expanded significantly for both the nonsevere and severe forms. The use of anti-inflammatory treatment, even for the mildest cases, and the withdrawal of symptomatic bronchodilation as monotherapy have been included in the most recent guidelines. Also, different biological therapies have revolutionized the therapeutical approach for severe uncontrolled asthma in children and adolescents.

**Summary:**

With the expanding landscape of novel therapeutic approaches for pediatric asthma, further evidence is needed to help clinicians choose the best option for patients, particularly those with severe asthma. The identification of novel predictive biomarkers may also help pediatricians in selecting children and adolescents for innovative therapies.

## INTRODUCTION

Asthma is a chronic inflammatory disease of the airways characterized by airway hyperresponsiveness, acute and chronic bronchoconstriction, airway edema, and mucus plugging. The inflammatory component of asthma involves many cell types, including mast cells, eosinophils, T lymphocytes, neutrophils, and epithelial cells and their biological products. For most asthma patients, a regimen of controller therapy and reliever therapy provides adequate long-term control [1^▪▪^]. Most children with asthma have the mild or moderate form and can obtain adequate control through the avoidance of triggering factors and/or with the help of medications, such as short-acting inhaled b2-receptor agonists (SABA), inhaled corticosteroids (ICS), and, when needed, the addition of long-acting b2-agonists (LABA) and leukotriene receptor antagonists (LTRA) [2^▪^]. However, 2–5% of all asthmatic children have uncontrolled asthma despite receiving maximum treatment with conventional medications, requiring additional biologic treatment [3,4].

Recently, the therapeutic tools for pediatric asthma have expanded significantly for both the nonsevere and severe forms. Thus, this review aims to summarize the most recent advances in asthma management, focusing on novel approaches to pediatric asthma. 

**Box 1:**
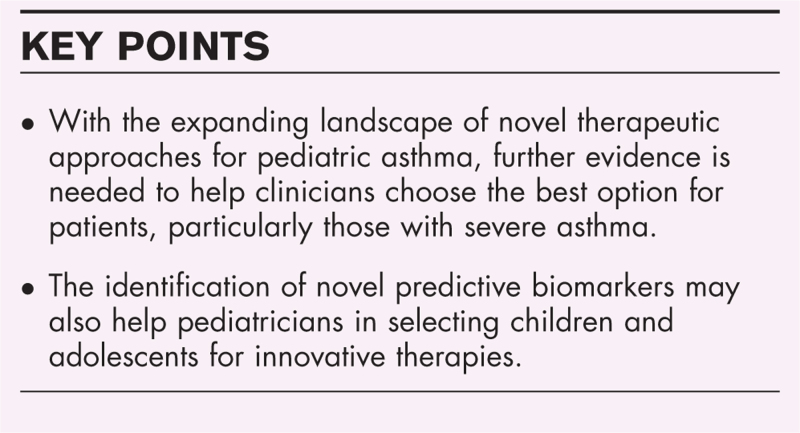
no caption available

## FOCUS ON RECENT ADVANCES IN MILD ASTHMA TREATMENT

In the pediatric age group, ‘intermittent’ and ‘mild-persistent’ account for the vast majority of asthma phenotypes [5–10]. As described in the British National Review of Asthma Deaths report in 2014, mild asthma is associated with a considerable risk of severe and, potentially, fatal acute attacks [11]. Some evidence shows that its usually uncomplicated nature, as well as the low frequency of symptoms in patients with mild asthma, are not only connected to poor adherence to controller therapy – specifically, ICS – but also to short-acting β2 agonist (SABA) abuse with subsequent adverse events (headache, tremors, and tachycardia above all) [7,9].

In 2019, GINA implemented a revolutionary strategy to treat mild and intermittent asthma [12,13].

The main modifications concerned adult and adolescent patients, especially those cases of unsatisfactory adherence to controller therapy. As the first step of asthma therapy, treatment based exclusively on isolated SABA was replaced with ‘ICS taken whenever SABA is taken’ and low, symptom-driven doses of ICS-formoterol (as needed) [1^▪▪^,^12^,^13^].

The effectiveness of these options in adolescents and adults in reducing unplanned healthcare visits, hospitalizations, or exacerbations, as well as exposure to systemic corticosteroids, has been demonstrated in the literature. In addition, their probable effectiveness in reducing adverse events has been reported in the literature [14]. Nonetheless, no such evidence is available among children under the age of 12. To compensate for such lack, a parallel-group, open-label, 52-week, phase III randomized clinical trial (RCT) is currently recruiting children aged 5–15 years with mild asthma. The goal is to assess the safety and efficacy of as-needed budesonide-formoterol compared with as-needed salbutamol [15]. The results will be helpful for evidence-based treatment for mild pediatric asthma.

For children aged 6–11 years with the intermittent form, the 2022 update of the GINA document calls for the use of low-dose ICS together with SABA (step 1) and, in mild persistent cases, a daily low dose of ICS (step 2) [1^▪▪^,^8^]. As Sumino *et al.*[16] have recently proved, in primary care settings, the symptom-based, intermittent use of ICS is a valuable, patient-centered strategy for mild pediatric asthma. ICSs are considered to be well tolerated and effective in preventing exacerbations, improving pulmonary function, and enabling rescue medication utilization and asthma control in members of this age group with persistent symptoms [17]. Treatment with ICS is generally well tolerated in children at the recommended dosages, even when taken for prolonged periods. However, side effects because of local deposition in the oropharynx and larynx (e.g. dysphonia and local candidiasis) and/or systemic side effects (e.g. adrenal suppression, ocular, and skeletal effects) may occur [18,19]. ICS therapy may be associated with a deceleration of growth velocity in children with persistent asthma, particularly at higher doses. The risk of systemic side effects from ICS may depend on the drug molecule, cumulative dose, delivery system, individual differences in response to the glucocorticoid, and the degree to which the drug is absorbed in different sites [18,19]. Despite their reduced effectiveness compared with ICS, daily LTRA may be considered another option [20]. The administration of ICS and other controller therapies should be determined through different phases, according to the frequency of symptoms. In addition, the use of ICS at the beginning of a treatment represents an option in the presence of validated practical tools, such as poor lung function, or increased levels of total IgE, eosinophils, or fractional exhaled nitric oxide (FeNO) [1^▪▪^].

Whenever an additional therapy is necessary, instead of adding LTRA or increasing the dose of ICS, ICS can be integrated with LABA as the first option with proven efficacy [21,22]. Otherwise, a daily, as-needed and very-low dose of ICS-formoterol can be considered as an option for step 3 [1^▪▪^,^23^,^24^]. The regular use of LABA alone has been associated to safety concerns in the past [25]. A Cochrane review carried out by O'Shea *et al.*[26] has recently assessed the risk of mortality and nonfatal but serious adverse reactions associated with regular use of salmeterol-ICS and formoterol-ICS in asthma treatment. No data regarding safety problems emerged from the two retrieved studies conducted among the pediatric population, although further research in this area remains necessary. New interesting data may possibly also come from current pediatric studies assessing the combination of an ICS and formoterol as an option for both quick relief and maintenance therapy [15]. In real life, clinicians should make personalized, case-driven choices between formoterol and salmeterol combination inhalers in the course of regular pediatric maintenance therapy.

Finally, allergen immunotherapy (AIT) at present represents the unique disease-modifying treatment strategy for IgE-mediated allergic diseases [27]. AIT can induce clinical improvement of allergic asthma, including reduced symptoms, medication use, and improvement of quality of life, with a long-lasting effect after cessation of treatment [28]. Notably, the current asthma guidelines now recommend sublingual immunotherapy as an add-on therapy for asthma in adults and adolescents with house dust mite allergy [28]. Clinical indications of AIT, with particular reference to asthma, mechanisms of immunological tolerance to allergens, and the potential biomarkers predicting clinical response represent active research fields in pediatric asthma treatment [29].

## SEVERE ASTHMA MANAGEMENT IN CHILDREN

**FIGURE 1:**
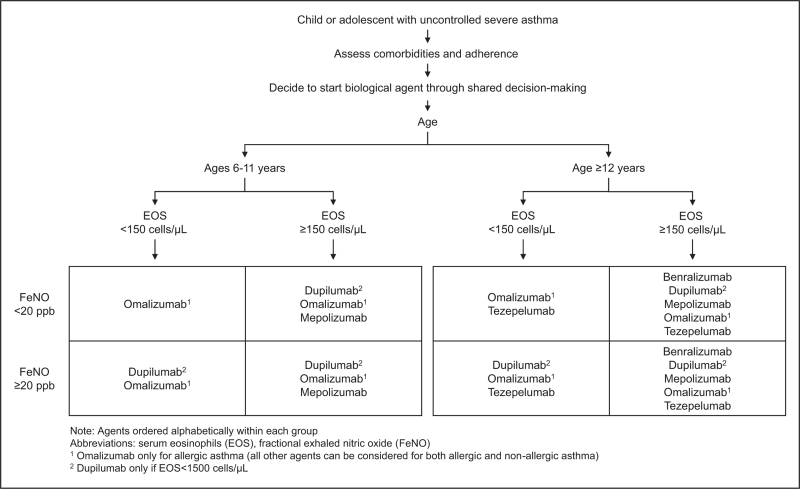
Suggested algorithm for selecting a biologic agent for pediatric severe asthma. Note: Agents ordered alphabetically within each group. EOS, serum eosinophils; FeNO, fractional exhaled nitric oxide. ^1^Omalizumab only for allergic asthma (all other agents can be considered for both allergic and nonallergic asthma). ^2^Dupilumab only if EOS less than 1500 cells/μl.

In recent years, various highly effective add-on therapies have been developed for treating severe asthma, including monoclonal antibodies targeting type 2 inflammatory pathways. These biological therapies are now recommended as first-line add-on treatment choices among the pediatric population as well (Table 1) [30]. These therapies have been shown to be effective and well tolerated. With this expanding landscape of available biologics for children and adolescents, choosing one can be challenging. Recently, Saxena *et al.* proposed a selection algorithm based on a patient's asthma phenotype and biomarkers (Fig. 1) [3].

**Table 1:** Clinical trials with approved biologics in children with uncontrolled severe asthma

	Study characteristics	Efficacy outcome	Safety outcome	Reference
Omalizumab				
Efficacy and Safety of Omalizumab for the Treatment of Severe or Poorly Controlled Allergic Diseases in Children Systematic Review Registration: (https://www.crd.york.ac.uk/PROSPERO), identifier (CRD42021271863)	Systematic review and meta-analysis of randomized controlled trials (RCTs) on the efficacy and safety of omalizumab in children with severe asthma	Omalizumab may reduce exacerbations of severe asthma at 12 weeks [risk ratio (RR), 0.52; 95% confidence interval (CI) 0.31–0.89], 24 weeks (RR, 0.69; 95% CI 0.55–0.85; GRADE: moderate-quality evidence) and 52 weeks (RR, 0.62; 95% CI 0.40–0.94; GRADE: moderate-quality evidence) as well as the dose of inhalation corticosteroid compared with placebo	Good safety profile. Although the incidence of SAEs was 3.6–13.7%, only three cases were judged to be related to omalizumab, including one case of generalized urticaria, one case of moderate tic disorder and one case of anaphylaxis 10 h after the third injection	[35]
Mepolizumab								
MUPPITS-2 ClinicalTrials.gov number, NCT03292588	Phase 2 290 children aged 6–17 y Mepolizumab 40 mg and 100 mg s.c. vs. placebo q4w 52-week treatment period	The mean number of asthma exacerbations within the 52-week study period was 0.96 (95% CI 0.78–1.17) with mepolizumab and 1.30 (1.08–1.57) with placebo (rate ratio 0.73; 0.56–0.96; p = 0.027). No between-group differences in FEV1% predicted, FEV1/FVC, or measures of impulse oscillometry. Improvement of CASI from baseline in both the mepolizumab and placebo groups with no significant difference between treatment groups.	Frequency of AEs: 29% with mepolizumab and 11% with placebo Common AEs in the mepolizumab group: injection site reactions; skin and subcutaneous disorders, gastrointestinal disorders, nervous system disorders (e.g. headache, dizziness, syncope)	[49]
Benralizumab				
BORA ClinicalTrials.gov number, NCT02258542	Safety extension study 86 adolescents aged 12–17 y Patients on benralizumab 30 mg every 4 weeks (q4w) or every 8 weeks (q8w) in SIROCCO/CALIMA continued their regimens in BORA for 108 weeks	69% Q8W patients were exacerbation-free. Mean change in FEV1 at week 108 versus baseline was greater in Q8W vs Q4W groups	For q4w and q8w regimens, the rates of TEAEs were 68% and 74%; TEAEs leading to discontinuation were 4% and 0%, serious AEs were 8% and 7%. No deaths occurred.	[55]
Dupilumab				
Liberty Asthma VOYAGE ClinicalTrials.gov number, NCT02948959	Phase 3 408 children aged 6–11 y dupilumab 100 and 200 mg s.c. vs. placebo q2w 52-week treatment period	Annualized rate of SEA: 0.31 (95% CI 0.22–0.42) with dupilumab and 0.75 (95% CI 0.54–1.03) with placebo ppFEV1: mean change from baseline 10.5 ± 1.0 percentage points with dupilumab and 5.3 ± 1.4 percentage points with placebo Significantly better asthma control than placebo (*P* < 0.001)	Frequency of AEs: 83.0% with dupilumab and 79.9% with placebo Common AEs in the dupilumab group: viral infection of the upper respiratory tract; eosinophilia without clinical symptoms; parasitic infections; asthma exacerbations	[58]
Tezepelumab				
NAVIGATOR ClinicalTrials.gov number, NCT03347279	Phase 3 1061 patients aged 12–80 y (82 patients aged 12–17 y) Tezepelumab 210 mg s.c. vs. placebo q4w 52-week treatment period	Annualized rate of asthma exacerbations: 0.93 (95% CI 0.80–1.07) with tezepelumab and 2.10 (95% CI 1.84–2.39) with placebo (rate ratio, 0.44; 95% CI 0.37–0.53; *P* < 0.001)	The frequencies and types of adverse events did not differ meaningfully between the two groups	[65]

ACQ, Asthma Control Questionnaire; AEs, adverse events; C-ACT, children asthma control test; CASI, composite asthma severity index; CI, confidence interval; ppFEV1, predicted prebronchodilator forced expiratory volume in 1 s; q2w, every 2 weeks; q4w, every 4 weeks; SAEs, serious adverse events; s.c., subcutaneous; SEA, severe exacerbation events; y, years; TEAEs, treatment-emergent adverse events.

### Omalizumab

Omalizumab is the first-available humanized monoclonal antibody targeting IgE. In 2003, the medication was approved for the treatment of moderate-to-severe allergic asthma, and its indication was extended in 2016 to include children aged at least 6 years [31,32]. The data on its efficacy and safety have been previously discussed [32–34]. Recently, a meta-analysis evaluating a total of 2168 asthmatic children treated with omalizumab showed that it was effective in reducing the risk of asthma exacerbations [risk ratio 0.52, 95% confidence interval (CI) 0.31–0.89] [35]. Moreover, the dose of inhaled corticosteroids, the use of oral corticosteroids (OCS), and the need for additional rescue medication were lower in children treated with omalizumab compared with controls. These effects allowed for a better asthma control and an improved quality of life (QoL) in children and their families [36]. The steroid-sparing effect and the reduction in the exacerbation number in omalizumab-treated children have been confirmed by real-life studies [37–40].

Regarding safety, omalizumab is generally well tolerated. The main side effects reported have been local (pain at the injection site, skin reactions) and have had a short resolution [37–39]. Moreover, no clear increased risk of malignancy in patients treated with omalizumab has been reported [41,42], but long-term monitoring of treated patients is still required to confirm the good safety profile.

Definitive data on validated biomarkers predicting response to omalizumab treatment are still lacking and require further investigation. Omalizumab seems to be more effective in reducing asthma exacerbations in patients with low forced expiratory volume in the first second (FEV_1_) at baseline, allergic comorbidities, high IgE and blood eosinophil counts, and/or high FeNO [43].

### Mepoluzimab

Mepolizumab is a humanized mAb that targets free IL-5, a critical cytokine regulating eosinophil development, differentiation, trafficking, and survival [44]. The medication was first approved as an add-on treatment for adults and adolescents (≥12 years) with severe asthma with an eosinophilic phenotype (blood eosinophil counts ≥150 cells/μl) [35]. In this population, the results of the MENSA Phase 3 study demonstrated a reduction in severe asthma exacerbations by 53%, an improvement in markers of asthma control, and an improvement in FEV_1_ in the treated group relative to the placebo group [45]. A post hoc meta-analysis of the mepolizumab clinical trials that included adolescents showed comparable exacerbation rate ratios relative to the placebo group, as seen in adults (0.46 in adults, 0.60 in adolescents) but with substantially wider CIs (0.38–0.56 in adults, 0.17–2.10 in adolescents). It should be noted that a limited number of adolescents were included in these studies [46].

The approval of mepolizumab in children aged at least 6 years comes from the results of the phase 2 open-label study conducted in 36 children, which demonstrated pharmacokinetic and pharmacodynamic properties, as well as a safety profile, comparable to those seen in adults and adolescents [47,48]. Finally, a phase 2, randomized, placebo-controlled, 52-week study (MUPPITS-2) evaluated mepolizumab in patients aged 6–17 years with exacerbation-prone severe eosinophilic asthma living in US urban locations [49]. A 27% reduction in asthma exacerbations was observed in patients treated with mepolizumab compared with the placebo group (*P* = 0.027). However, no differences were observed between treatment groups in the time to the first exacerbation, asthma control, and lung function. No new safety concerns were identified in children aged 6–11 years compared with the known safety profile in patients aged at least 12 years [49].

### Benralizumab

Benralizumab is a humanized monoclonal antibody that targets the IL-5 receptor α chain (IL-5Rα), modulating eosinophilic inflammation [50–52]. In 2017, this biologic was approved as an add-on maintenance treatment in adolescents (12 years and older) and adults with severe eosinophilic asthma.

Benralizumab's efficacy and safety have been assessed through phase 3 randomized controlled trials [53–56]. Overall, benralizumab significantly reduced the annual asthma exacerbation rate compared with placebo, especially in patients with high baseline blood eosinophils (≥300 cells/μl), with a good safety profile.

Focusing on the pediatric patients included in the studies, 53 and 55 adolescents (aged 12–17 years) were included in the SIROCCO [53] and CALIMA [54] studies, respectively. Of note, the patients with blood eosinophils at least 300 cells/μl, the rate ratios for severe exacerbations relative to placebo were 1.57 (95% CI 0.13–13.96) in CALIMA and 1.77 (95% CI 0.40–7.78) in SIROCCO, pointing out a potential higher risk of exacerbation in the group treated with benralizumab. However, the limited number of patients included, and the broad CIs make these findings difficult to interpret. Interestingly, the BORA study evaluated the follow-up outcomes after 3 years of treatment for the adolescents enrolled in the SIROCCO and CALIMA trials [55]. The analysis of 69 patients who completed the study showed a low rate of asthma exacerbation, with 85.7% (on every 4-week regimen) and 75% (on every 8-week regimen) remaining exacerbation-free while continuing benralizumab over 108 weeks. In addition, benralizumab use during this time period appeared to be well tolerated.

### Dupilumab

Dupilumab is a recombinant human IgG4 antibody targeting the IL-4 receptor, inhibiting the biological effects of both IL-4 and IL-13 [57]. Dupilumab was approved for the treatment of asthma in patients 6–11 years of age based on a phase 3 VOYAGE efficacy and safety study: compared with placebo, dupilumab reduced the annualized rate of severe asthma exacerbations, improved lung function, and enhanced asthma control in children with uncontrolled, moderate-to-severe asthma with evidence of type 2 inflammation [as identified by blood eosinophils ≥150 cells/μl or FeNO ≥20 parts per billion (ppb]) [58]. Further analysis of the VOYAGE study assessed dupilumab pharmacokinetics and the effects on type 2 biomarkers [59]. Results revealed that the weight-tiered dose regimens for children (100 and 200 mg every 2 weeks) achieved mean concentrations within the dupilumab therapeutic range, and similar decreases in type 2 biomarker levels [serum total IgE, serum thymus and activation-regulated chemokine (TARC), blood eosinophil counts, and FeNO] [59].

In addition, dupilumab has been approved in older patients with asthma. In the pivotal Phase 3 QUEST study that included adults and adolescents with persistent asthma uncontrolled on ICE and a second bronchodilator, dupilumab at both 300 and 200 mg every 2 weeks led to a significant reduction in exacerbations and improvement in lung function compared with placebo [60]. This effect was significant for patients with evidence of type 2 inflammation including those with either a baseline blood eosinophil level at least 150 cells/μl and/or baseline FeNO level at least 25 ppb. In the pivotal Phase 3 VENTURE study in adults and adolescents with corticosteroid-dependent asthma, an improvement in lung function and reduction in exacerbations was seen in patients treated with dupilumab despite OCS taper and regardless of screening eosinophil levels [61]. These studies have confirmed an acceptable profile and a positive benefit–risk profile in adults and adolescents with moderate-to-severe asthma.

### Tezepelumab

Tezepelumab is a human monoclonal antibody that targets and blocks the thymic stromal lymphopoietin (TSLP). As an alarmin, TSLP, an airway epithelial-derived cytokine, can activate the type 2 inflammatory pathways [62]. Tezepelumab was approved in 2021 for adolescents (12 years and older) and adults with severe asthma, regardless of endotype or phenotype [3]. Five clinical trials evaluated the efficacy and safety of Tezepelumab [63–67].

Of note, only the NAVIGATOR trial included pediatric patients (aged 12–17 years) [65]. In this trial, participants were randomized to receive tezepelumab or a placebo and were followed for acute asthma exacerbations over 52 weeks. The tezepelumab-treated group showed a significantly lower rate of annual asthma exacerbations compared with the placebo group (rate ratio 0.44, 95% CI 0.37–0.53). Overall, tezepelumab was effective in improving asthma control and lung function, with positive effects on the quality of life. Moreover, tezepelumab therapy reduced blood eosinophils, IgE levels, and FeNO, suggesting a broad-spectrum effect in blocking type 2 inflammatory pathways. The results from this trial were comparable to those in other trials with only adults.

Regarding safety, tezepelumab was well tolerated, with the most frequent adverse effects noted to be arthralgias, back pain, and pharyngitis [68]. Of note, in a subgroup analysis limited to adolescents (*n* = 41), the rate ratio for severe exacerbations favored tezepelumab (0.70, 95% CI 0.34–1.46) but with a wide confidence interval, potentially because of the limited number of pediatric patients in this age group.

## CONCLUSION

Asthma is a chronic inflammatory disease that severely affects patients’ health and quality of life. Recently, new approaches to childhood asthma treatment have been developed with the aim of improving asthma control. The use of anti-inflammatory treatment, even for the mildest cases, along with the withdrawal of symptomatic bronchodilation as monotherapy, has been included in the most recent guidelines. Moreover, as omalizumab was approved for asthma management, different biological therapies have revolutionized the therapeutical approach to severe uncontrolled asthma in children and adolescents. However, several unmet needs should be urgently addressed. First, comparative studies are required to help clinicians choose the best therapeutic option for patients with severe asthma who are eligible for more than one treatment. Second, standardized algorithms for the management of severe pediatric asthma should be realized, as already available in adults. Third, identifying novel predictive biomarkers is a fundamental goal in asthma management that may help physicians identify and select children and adolescents with severe asthma for innovative biologic therapies. Fourth, the possible duration of these therapies in this age group, as well as their potential action on airway remodeling, should be clarified.

The improved knowledge from ongoing and future studies will allow for a more comprehensive application of the precision medicine approach. In this context, biological treatment may benefit not only patients with the most severe phenotype but also those with mild-to-moderate forms or it may even be used to prevent asthma before its development.

## Acknowledgements

*None.*

### Financial support and sponsorship

*None.*

### Conflicts of interest

*There are no conflicts of interest.*

## REFERENCES AND RECOMMENDED READING

Papers of particular interest, published within the annual period of review, have been highlighted as:

▪ of special interest
▪▪ of outstanding interest
